# Does Size Matter? Evaluating the Impact of Intermediate Screw Length in Short-Segment Fixation of Thoracolumbar A3–A4 Fractures

**DOI:** 10.3390/jcm15062221

**Published:** 2026-03-14

**Authors:** Andrea Perna, Andrea Franchini, Luca Ricciardi, Francesco Maruccia, Luca Macchiarola, Felice Barletta, Franco Gorgoglione, Giuseppe Rovere

**Affiliations:** 1 Department of Orthopedics and Traumatology, Fondazione Casa Sollievo della Sofferenza, IRCCS, 71013 San Giovanni Rotondo, Italy; a.franchini@operapadrepio.it (A.F.);; 2 Neurosurgery Unit, Department of NESMOS, Sapienza University of Rome, 00185 Rome, Italy; 3 Department of Orthopaedics and Traumatology, “Policlinico Tor Vergata” Foundation, 00133 Rome, Italy; 4 Department of Clinical Sciences and Translational Medicine, Section of Orthopaedics and Traumatology, University of Rome Tor Vergata, 00133 Roma, Italy; 5 Faculty of Medicine, Catholic University “Our Lady of Good Counsel”, 1000 Tirana, Albania

**Keywords:** thoracic vertebrae, lumbar vertebrae, spinal fractures, bone screws, internal fixators, treatment outcome

## Abstract

**Background**: Short-segment posterior fixation with intermediate pedicle screws is widely used for thoracolumbar junction (TLJ) burst fractures. However, the optimal penetration depth of intermediate screws remains controversial. The aim of this study was to evaluate whether intermediate screw penetration depth influences radiographic alignment and functional outcomes at 12 months following short-segment posterior fixation of AO Spine A3–A4 thoracolumbar burst fractures. **Methods**: This retrospective cohort study included 105 patients with AO Spine A3–A4 TLJ burst fractures treated between 1 January 2019 and 31 December 2022. All patients underwent short-segment posterior stabilization with intermediate screws at the fracture level. Penetration depth was categorized as either <50% (Group A) or ≥50% (Group B) of vertebral body depth. Radiographic parameters (kyphotic deformity, segmental kyphosis, sagittal index, anterior vertebral body height) and clinical outcomes (Visual Analog Scale and Oswestry Disability Index) were evaluated preoperatively and at 12 months. **Results**: Both groups demonstrated significant postoperative improvement in radiographic alignment and clinical outcomes. No statistically significant differences were detected between groups in kyphotic correction, loss of correction, pain reduction, disability scores, operative time, length of stay, or complication rates at 12 months. **Conclusions**: Within the limitations of this retrospective study, intermediate screw penetration depth did not significantly influence radiographic or clinical outcomes at 12 months. Screw length selection may therefore depend on anatomical considerations and surgeon preference rather than expected differences in clinical performance.

## 1. Introduction

Fractures of the thoracolumbar junction (TLJ, T11–L2) represent the most frequently involved region in traumatic vertebral injuries, largely due to the unique biomechanical transition between the rigid thoracic spine and the more flexible lumbar region [1,2]. Among these injuries, burst fractures—classified as A3 and A4 according to the AO Spine system—are particularly common and often require surgical management [3].

According to the AO Spine thoracolumbar classification system, type A3 fractures are defined as incomplete burst fractures involving a single endplate with posterior wall involvement, whereas type A4 fractures represent complete burst injuries affecting both endplates and the posterior vertebral wall. These fractures result from axial compression and are characterized by varying degrees of vertebral body comminution, frequently associated with compromise of sagittal alignment.

In younger individuals, thoracolumbar burst fractures are typically related to high-energy trauma, such as motor vehicle accidents or falls from height. In contrast, in elderly populations, low-energy mechanisms and fragility-related injuries are increasingly observed due to population aging and reduced bone mineral density [1,2].

Despite the availability of various operative strategies—including open fixation, minimally invasive techniques, and combined anterior–posterior approaches—posterior pedicle screw instrumentation remains the most widely adopted technique because of its reliability and favorable clinical outcomes [4,5]. Short-segment posterior fixation, involving stabilization of one vertebra above and below the fracture, is commonly preferred as it preserves motion segments and reduces surgical morbidity. However, concerns remain regarding potential loss of reduction and late kyphotic deformity, particularly in unstable burst fractures [4,5,6].

To enhance construct stability, the addition of intermediate screws placed directly into the fractured vertebra has been increasingly advocated. Biomechanical and clinical studies have demonstrated that this configuration improves construct stiffness, facilitates fracture reduction through ligamentotaxis, and reduces postoperative kyphosis, achieving outcomes comparable to longer constructs while preserving spinal mobility [7,8,9,10,11].

Nevertheless, several technical aspects remain insufficiently defined, including the optimal diameter and, more specifically, the ideal length or penetration depth of intermediate screws. While previous studies have evaluated the presence or absence of intermediate screws or compared different screw designs, limited evidence specifically addresses screw length as an independent variable [9,10,11]. Only one prior study has directly investigated outcomes in relation to screw size, and no clear consensus has been reached regarding whether shorter or longer intermediate screws provide measurable clinical or radiological advantages.

Unlike previous investigations, the present study specifically evaluates the effect of intermediate screw length (relative penetration < 50% versus ≥50% of vertebral depth) on radiographic alignment, construct stability, and functional outcomes at 12 months in patients with AO Spine A3–A4 TLJ burst fractures.

A 50% vertebral body penetration threshold was selected, as previously described in clinical and biomechanical research, to distinguish screws engaging only the posterior half of the vertebral body from those extending into the anterior half, which theoretically provide greater anchorage and stability. Rather than assuming equivalence, this study was designed to detect any statistically significant differences in outcomes at 12-month follow-up.

The aim of this study was to determine whether intermediate screw penetration depth influences radiographic alignment, construct stability, and functional outcomes at 12 months following short-segment posterior fixation of AO Spine A3–A4 thoracolumbar burst fractures.

## 2. Materials and Methods

### 2.1. Research Design

This retrospective study analyzed patients treated surgically for vertebral burst fractures (VBFs) classified as A3 to A4 at TLJ between 1 January 2019 to 31 December 2022., according to the STROBE guidelines [12] (Table 1). This study was not designed as an equivalence or non-inferiority trial. Clear inclusion and exclusion criteria were predefined to ensure methodological consistency and reproducibility. Eligible patients were those presenting with single-level AO Spine A3–A4 thoracolumbar junction burst fractures treated with short-segment posterior fixation including intermediate screws, without neurological deficits, and with a minimum follow-up of 12 months. Patients with osteoporotic fractures (T-score ≤ −2.5), pathological fractures, prior spinal surgery, concurrent spinal injuries, severe head trauma, or use of polymethyl methacrylate augmentation were excluded. Detailed criteria are reported in Section 2.7.

### 2.2. Ethical Considerations

The study was conducted in accordance with the ethical principles of the Declaration of Helsinki (1964) and its subsequent amendments, including those adopted by the 75th World Medical Association (WMA) General Assembly (Finland, 19 October 2024). All participants provided written informed consent for using their clinical data in research projects. The Institutional Review Board stated that formal ethical approval was unnecessary, especially given partial data overlap with prior approved studies. No patients from this cohort were included in prior publications except for aggregate baseline data used in an institutional retrospective database study; no overlapping analyses were published.

### 2.3. Database Development

Collected data included demographic information, clinical and radiographic out-comes, neurological status assessed via the Frankel score [13], fracture classification based on AO Spine guidelines [3], surgery duration, hospital length of stay (LOS), and pain evaluations using a visual analog scale. The authors retrieved follow-up data from a clinical assessment at 1, 3, 6, and 12 months. The mean age of the cohort (approximately 56 years) makes severe osteoporosis unlikely except in selected individuals, thereby reducing the probability of major systematic differences in bone quality between groups.

All fractures were classified as AO Spine A3 or A4, which by definition involve the posterior vertebral wall. Consequently, posterior wall injury was homogeneous between groups and was not considered a differentiating variable. Although mean clinical follow-up exceeded 24 months, 12-month imaging was available for all included patients and was therefore chosen as the primary radiological endpoint to avoid attrition bias.

### 2.4. Study Group Formation

**Figure 1:**
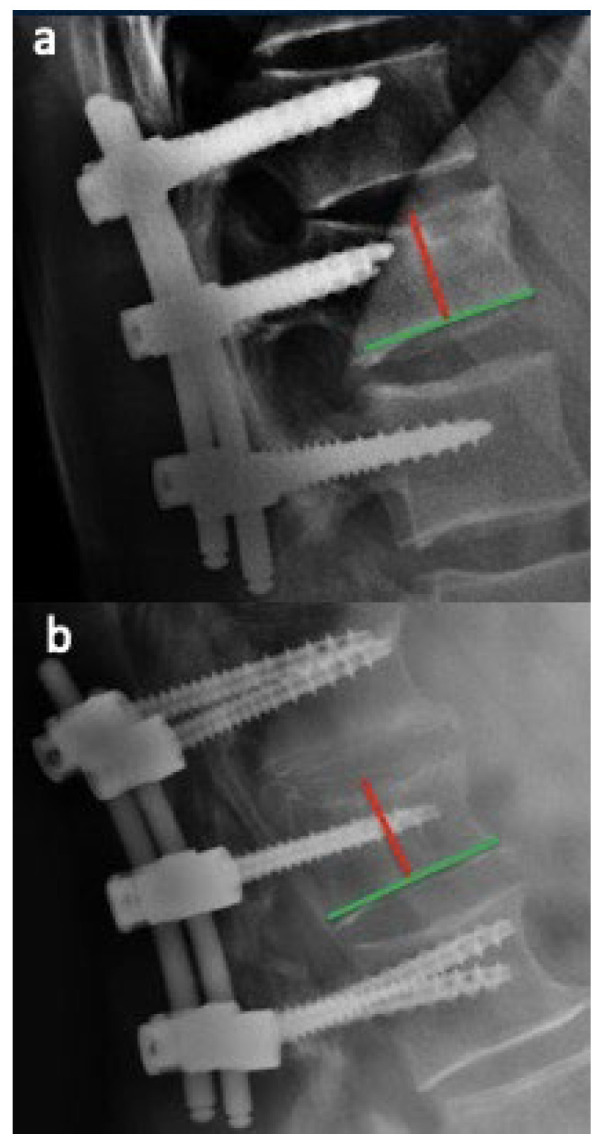
Postoperative radiographs of patients from both groups are shown. In subfigure (**a**), a patient from Group A is presented, with the intermediate screw not exceeding 50% of the vertebral body length. In subfigure (**b**), a patient from Group B is shown, where the intermediate screw clearly extends beyond 50% of the vertebral body length.

**Figure 2:**
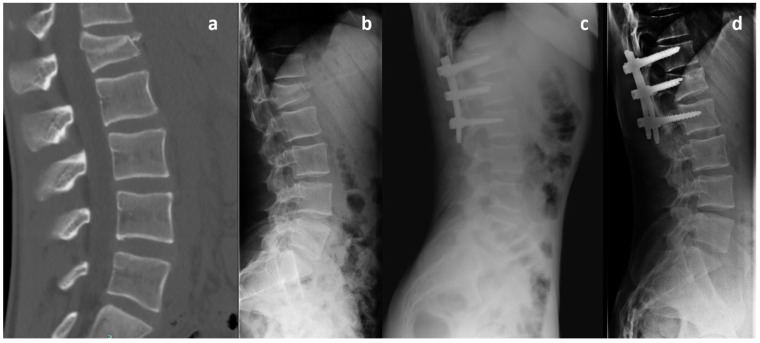
A 38-year-old female patient with an L1 A3 burst fracture included in Group A (short intermediate pedicle screws). (**a**) Preoperative CT scan showing kyphotic deformity and posterior wall involvement of the L1 vertebral body. (**b**) Preoperative lateral plain radiograph. (**c**) Immediate postoperative lateral radiograph demonstrating satisfactory correction of kyphotic deformity and restoration of anterior vertebral body height. (**d**) Lateral radiograph at 12-month follow-up showing good maintenance of correction with no signs of screw loosening or implant failure. Notably, the intermediate screws placed in the fractured vertebra are short, not exceeding 50% of the vertebral body length.

**Figure 3:**
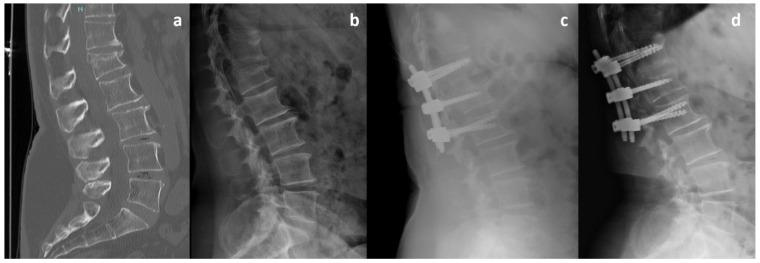
A 23-year-old male patient with an L1 A3 burst fracture included in Group B (Long intermediate pedicle screws). (**a**) Preoperative CT scan showing kyphotic deformity and posterior wall involvement of the L1 vertebral body. (**b**) Preoperative lateral plain radiograph. (**c**) Immediate postoperative lateral radiograph demonstrating satisfactory correction of kyphotic deformity and restoration of anterior vertebral body height. (**d**) Lateral radiograph at 12-month follow-up showing good maintenance of correction with no signs of screw loosening or implant failure. Notably, the intermediate screws placed in the fractured vertebra are long, exceeding 50% of the vertebral body length.

**Figure 4:**
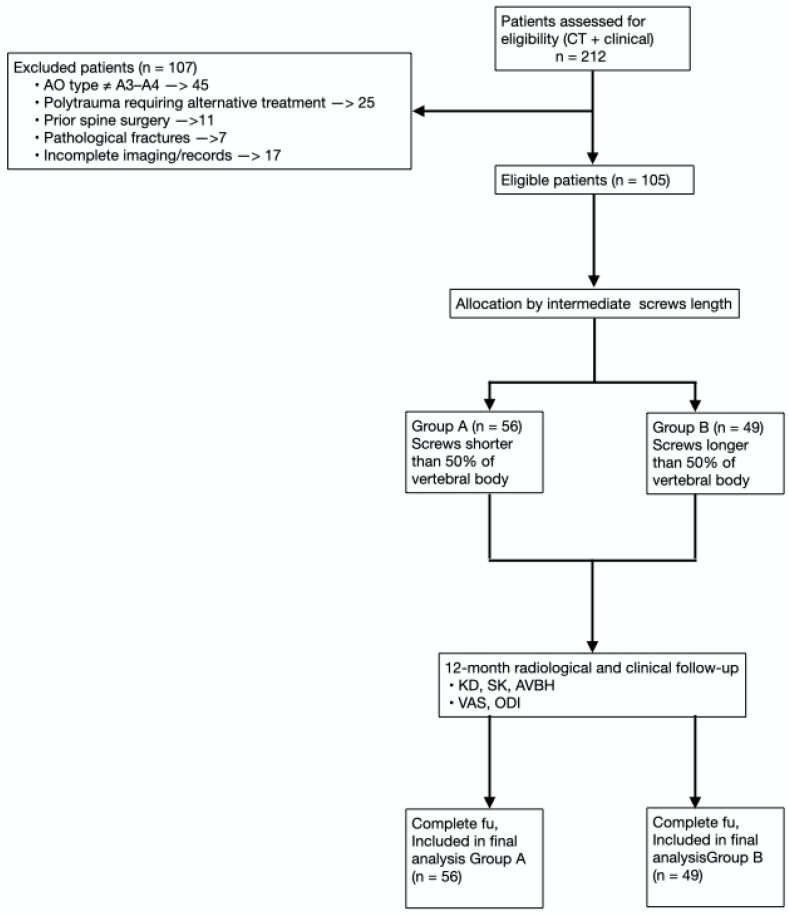
Patient flow diagram illustrating the screening, eligibility evaluation, exclusions, and final group allocation.

Patients were categorized into two groups based on the relative screw penetration ratio on lateral radiographs. Penetration ratio was defined as: screw tip distance from the posterior vertebral wall divided by sagittal vertebral body depth (mm), measured using PACS with built-in magnification correction. In Group A, the additional screws have a length of less than 50% of the vertebral body, ranging between 35 and 40 mm. In Group B, the intermediate screws have a length exceeding 50% of the vertebral body and measuring 45 mm or more. In all cases, the screw diameter was at least 6 mm (Figure 1). Representative images of patients from Group A and Group B are shown in Figure 2 and Figure 3, respectively. A graphical patient flow diagram was reported in Figure 4.

### 2.5. Radiological Assessment

All patients underwent spinal CT scans and standing lateral radiographs at admission. Follow-up evaluations were performed using standardized standing lateral radiographs. The following radiographic parameters were retrospectively assessed:

Kyphotic Deformity (KD): Defined as the Cobb angle between the superior and inferior endplates of the fractured vertebra.

Segmental Kyphosis (SK): Measured as the angle between the superior endplate of the vertebra above and the inferior endplate of the fractured vertebra.

Anterior Vertebral Body Height (AVBH): Measured in millimeters on lateral imaging at the anterior aspect of the fractured vertebral body.

Sagittal Index (SI): Calculated as kyphotic deformity corrected for the normal sagittal contour (SNC) at the corresponding spinal level, according to the method described by Farcy et al. [14]. Specifically, SI was calculated as SI = KD − SNC.

Vertebral body depth was measured on midsagittal CT reconstructions as the linear distance between the posterior and anterior cortical walls at the mid-vertebral level.

All radiographic measurements were independently performed by three senior spine surgeons blinded to group allocation. Inter-rater reliability was assessed using a two-way random-effects intraclass correlation coefficient (ICC) with absolute agreement. The ICC was 0.92 (95% CI 0.88–0.96), indicating excellent reliability.

### 2.6. Clinical Evaluation and Complications

Clinical outcomes were assessed using the Oswestry Disability Index (ODI) and the Visual Analog Scale (VAS) for back pain. The ODI is a validated patient-reported outcome measure specifically developed to quantify disability related to low back pain, as originally described by Fairbank and Pynsent [15]. The VAS is a widely adopted and validated instrument for pain intensity assessment, based on the method described by Huskisson [16]

ODI scores range from 0 to 100, with higher scores indicating greater disability. The VAS was recorded using a 10-point numerical scale, where 0 indicates no pain and 10 represents the worst imaginable pain.

Perioperative and follow-up complications were retrieved from medical documentation. Complication rates are reported with corresponding 95% confidence intervals.

### 2.7. Inclusion and Exclusion Criteria

Inclusion criteria were: (I) thoracolumbar junction (TLJ) fracture type A3–A4 according to the AO Spine classification system; (II) short-segment posterior stabilization with two screws in the fractured vertebra and in the adjacent vertebrae; (III) absence of neurological deficits; (IV) surgery performed within 72 h from admission; and (V) minimum follow-up of 12 months after surgery.

Exclusion criteria were: (I) infectious, malignant, or rheumatic vertebral conditions; (II) severe head trauma; (III) prior spinal surgery; (IV) concurrent spinal fractures; and (V) use of polymethyl methacrylate augmentation.

Bone mineral density was not systematically assessed using dual-energy X-ray absorptiometry (DEXA) scans. Although no patients had a documented history of metabolic bone disease in the available medical records, formal exclusion of osteoporosis based on standardized densitometric criteria was not performed.

### 2.8. Surgical Technique

Surgical treatment was indicated for preoperative SI > 15°. Hyperextension positioning on the operating table was initially used to partially correct segmental kyphosis (SK). Pedicle screws were placed in the vertebra above, the vertebra below, and the fractured vertebra using a free-hand transpedicular technique under fluoroscopic guidance.

All constructs consisted of a standardized six-screw configuration: two pedicle screws in the vertebra above, two in the vertebra below, and two intermediate screws in the fractured vertebra. Screw trajectory followed a conventional transpedicular orientation. Bicortical penetration was deliberately avoided in all cases. Screw diameter ranged from 6.0 to 7.0 mm depending on pedicle morphology, without systematic differences between groups. Therefore, screw number, orientation, and diameter were consistent variables across the study population.

Ligamentotaxis, rod contouring, and compression maneuvers were subsequently performed to further correct kyphosis. Zygapophyseal joints were sacrificed, and laminae were decorticated to promote posterior fusion.

The implant system used was either CD Horizon Solera (Medtronic) or Expedium (DePuy Synthes), depending exclusively on institutional availability. Both systems consist of titanium alloy pedicle screws coupled with 5.5 mm rods and polyaxial screw heads allowing multidirectional rod insertion and angular adjustment. Locking caps secure the rod–screw interface to create a rigid posterior construct. No expandable screws, cement augmentation, or alternative fixation systems were used. Implant system was included as a covariate in the adjusted mixed-effects model to control for potential confounding.

Group allocation was based on relative intermediate screw penetration depth, defined as <50% or ≥50% of the vertebral body anteroposterior diameter measured intraoperatively. Absolute screw length in millimeters (typically 35–40 mm for shorter screws and ≥45 mm for longer screws) is reported for descriptive purposes only and may vary according to vertebral body size. The primary grouping variable for statistical analysis was percentage penetration rather than absolute screw length.

The choice of intermediate screw length did not depend on fracture morphology or patient-related characteristics. Instead, it followed long-standing team-specific surgical protocols: in one spine surgery team, longer intermediate screws were routinely utilized, whereas the second team systematically adopted shorter screws. All procedures were performed by senior surgeons with more than 30 years of spine surgery experience. While this approach minimized intra-team variability in screw selection, it introduces a potential risk of confounding related to surgeon-specific practice patterns. Screw length selection was therefore adjusted for in the mixed-effects statistical models.

Antibiotic prophylaxis included ceftriaxone (2 g) and teicoplanin (400 mg) administered 60 min before skin incision.

### 2.9. Postoperative Care

Postoperative management followed a standardized institutional protocol. Patients were mobilized on postoperative day two under physiotherapist supervision, beginning with assisted standing and progressive ambulation as tolerated. A rigid three-point thoracolumbar brace was prescribed for continuous use during the first three postoperative months, except during hygiene and wound care. Gradual return to daily activities was allowed after the first postoperative month, while avoidance of heavy lifting and high-impact activities was recommended for at least three months.

Wound inspection and dressing changes were performed every three days until suture removal, which occurred approximately 14 days after surgery. Clinical and radiographic follow-up evaluations were scheduled at 1, 3, 6, and 12 months postoperatively. Rehabilitation exercises focused on progressive trunk stabilization and core strengthening after brace discontinuation.

### 2.10. Outcomes

Primary outcomes included the correction of kyphosis (KD) and loss of correction at 12 months. (ΔKD, ΔSK, ΔSI reported with means ± SD and 95% CI; study is exploratory and not powered for equivalence). Secondary outcomes encompassed clinical and functional scores, surgical duration, failure rates, and implant integrity. Effect sizes (Hodges–Lehmann median difference, rank-biserial effect size) and 95% CIs are reported; multi-plicity for primary outcomes controlled with Benjamini–Hochberg FDR.

### 2.11. Statistical Analysis

Ordinal variables: Mann–Whitney U-test (independent) and Wilcoxon Signed-Rank Test (dependent). Normality assessed by Kolmogorov–Smirnov test. Results reported as medians (IQR) for non-normal variables, means ± SD. In-ter-rater reliability: two-way random-effects ICC (absolute agreement). Primary outcomes adjusted for baseline deformity, AO type, fracture level, implant system, screw diameter, time to surgery; mixed-effects models included surgeon as random effect. To quantify the magnitude of differences between groups, we calculated standardized effect sizes for continuous variables. For normally distributed data, Cohen’s d was computed as the difference between group means divided by the pooled standard deviation. Effect sizes were interpreted as negligible (d ≈ 0.2), medium (d ≈ 0.5), or large (d ≥ 0.8). For variables not normally distributed, non-parametric effect sizes were estimated using the Hodges–Lehmann median difference and rank-biserial correlation. Reporting effect sizes complements *p*-values by providing an estimate of the practical significance of observed differences. No a priori sample size calculation or non-inferiority margin was defined for this study, as the analysis was retrospective and based on all eligible patients identified during the study period. To assess the precision of the estimates obtained, a post hoc precision analysis was performed. Given the total sample size and the observed variability of kyphotic deformity (KD), the study allows estimation of between-group differences with a statistical precision of approximately ±2.1° (95% confidence interval). This value reflects the width of the confidence interval and does not represent a predefined margin of clinical relevance or non-inferiority threshold.

This precision estimate reflects the accuracy of the observed between-group comparisons but does not imply that the study was powered to formally test equivalence or non-inferiority. Significance set at *p* < 0.05. Post hoc precision analysis: With the current sample, be-tween-group differences in KD can be estimated within ±2.1° (95% CI). Complete-case analysis was performed because no missing data were present for primary or secondary outcomes. Statistical analyses were performed using IBM SPSS Statistics for Windows, Version 26.0 (IBM Corp., Armonk, NY, USA)

## 3. Results

### 3.1. Study Population and Group Allocation

A hundred and five patients out of 212 retrieved from the database search were enrolled in the present study according to the inclusion criteria. Patients were divided into two groups based on the relative pedicle screw penetration ratio on lateral X-ray views. Patients with screws not exceeding 50% of the vertebral body were assigned to Group A (56 patients), while those with screws exceeding 50% of vertebral body length were as-signed to Group B (49 patients). Patient demographics are summarized in Table 2. No statistically significant difference was detected between groups in terms of age, sex, trauma mechanism, AO classification, or fracture level (all *p* > 0.05).

### 3.2. Radiographic Analysis

Inter-rater reliability (IRR) for radiographic measurements was assessed using a two-way random-effects ICC (absolute agreement), ICC = 0.92, 95% CI 0.88–0.96, with three raters blinded to group allocation. Preoperative values of KD, AVBH, SK, and SI showed no statistically significant difference between groups. As shown in Table 3, both groups exhibited significant postoperative improvements in KD, AVBH, SK, and SI compared to preoperative values. In Group A, KD improved from 17.4° (±5.1) to 3.2° (±4.2) (*p* < 0.0001), while in Group B, it improved from 18.1° (±4.3) to 3.6° (±6.1) (*p* = 0.0002). The between-group mean differences at 12 months with 95% confidence intervals were as follows: ΔKD 0.4° (95% CI −1.2 to 2.0), ΔSK 0.6° (95% CI −1.5 to 2.7), ΔSI 0.3° (95% CI −1.4 to 2.0), ΔAVBH 0.1 mm (95% CI −0.5 to 0.7), indicating no statistically significant differences between groups. Overall, the achieved corrections were maintained throughout the 12-month follow-up. All statements are reported as no statistically significant difference detected.

### 3.3. Operative Parameters and Hospitalization

The mean surgical time was 92 min (±23) in Group A and 94 min (±18) in Group B with no statistically significant difference detected between groups. Similarly, the number of fluoroscopic images taken during surgery, as detailed in Table 4, did not differ significantly between the groups. Length of hospital stay was comparable between the groups. Cohen’s d for surgical time was −0.10, indicating a negligible effect size, and no statistically significant difference was detected between groups. The mean number of fluoroscopic images was 6.3 ± 2.4 in Group A and 6.9 ± 3.1 in Group B, with Cohen’s d = −0.20, indicating a small effect size and no statistically significant difference detected. Length of hospital stay was 4.2 ± 1.1 days in Group A and 4.7 ± 0.8 days in Group B, with Cohen’s d = −0.52, indicating a medium effect size; however, this difference was not statistically significant. Overall, operative parameters and hospitalization outcomes were comparable between groups.

### 3.4. Pain and Functional Outcomes

Both groups demonstrated a significant reduction in VAS pain scores within the first postoperative month, with sustained improvement at subsequent follow-up assessments. ODI scores similarly improved over time in both cohorts. No statistically significant between-group differences were detected at any evaluated timepoint (Table 5).

The magnitude of postoperative improvement in VAS and ODI exceeded commonly reported minimal important difference (MID) thresholds (approximately 1–2 points for VAS and 10 points for ODI). However, the study was not designed as a non-inferiority or equivalence trial, and therefore no conclusions regarding comparative clinical equivalence can be drawn.

All patients presented with Frankel grade E at admission in accordance with the inclusion criteria. Neurological status remained stable throughout follow-up, and no new neurological deficits were observed in either group.

### 3.5. Complications

Two cases of screw mobilization occurred in Group A (2/56, 3.6%, 95% CI 0.4–12.2%) and one in Group B (1/49, 2.0%, 95% CI 0.05–10.5%). Both groups reported one case of implant failure (Group A: 1/56, 1.8%, 95% CI 0.05–9.6%; Group B: 1/49, 2.0%, 95% CI 0.05–10.5%) and one case of superficial wound infection (Group A: 1/56, 1.8%, 95% CI 0.05–9.6%; Group B: 1/49, 2.0%, 95% CI 0.05–10.5%). All infections were successfully managed with antibiotic therapy and did not require surgical revision. No statistically significant differences were detected between groups for any complication.

## 4. Discussion

### 4.1. Purpose

This study aimed to assess whether intermediate screw length (<50% vs. >50% vertebral depth) influences radiographic alignment, construct integrity, and functional rehabilitation after short-segment posterior fixation of A3–A4 TLJ fractures. In our cohort, both screw strategies caused a significant improvement, and no statistically significant differences were detected at 12 months; thus the study provides exploratory evidence that screw length alone did not materially affect the assessed outcomes.

### 4.2. Background

The management of burst fractures at the thoracolumbar junction (TLJ) remains controversial, with no universally accepted evidence-based guidelines available to date [14]. Treatment goals include restoring spinal stability, limiting kyphotic deformity, protecting neural structures when necessary, and enabling early mobilization. Posterior pedicle screw fixation is widely adopted due to its favorable safety profile, shorter operative time, and strong biomechanical performance [4,5,17,18]. Short-segment posterior fixation, involving pedicle screws placed one level above and below the fracture, offers several advantages such as motion-segment preservation, reduced blood loss, and lower morbidity compared to longer constructs [4,18]. Biomechanical studies demonstrate that inserting pedicle screws into the fractured vertebra significantly improves construct stability, fracture reduction, and minimizes loss of correction [4,10]. These outcomes are comparable to those achieved with long-segment stabilization or combined anterior–posterior approaches [10,18,19,20]. Despite this growing evidence, the use of intermediate screws in the fractured vertebra remains a subject of debate in the clinical community, mainly due to the lack of standardized guidelines regarding optimal screw characteristics. Nonetheless, increasing evidence supports the clinical efficacy of placing screws into the fractured vertebra, demonstrating improved biomechanical stability, reduced im-plant-related complications, and better preservation of sagittal alignment compared to constructs without intermediate screws [5,7,10,18,19]. Importantly, patient factors such as overall health status must guide the choice of surgical strategy. For example, in individuals with poor systemic conditions (e.g., spinal metastases), minimally invasive percutaneous approaches are preferred to minimize surgical burden, as recently con-firmed in a meta-analysis [21]. While the beneficial role of intermediate screws in KD, AVBH, SK, SI has been established, the optimal length and diameter of these screws remain undefined. Some authors have advocated for shorter screws in the fractured vertebra to minimize the risk of anterior vertebral wall perforation [22], whereas others favor screws of comparable size to those used in adjacent intact vertebrae [23]. A recent systematic review and meta-analysis evaluating 28 studies highlighted substantial heterogeneity and noted that no work to date has clearly defined optimal screw dimensions [24]. To date, only one study has specifically investigated the influence of intermediate screw size on postoperative outcomes [17]. In a cohort of 36 patients treated with short-segment posterior fixation, the authors compared shorter screws (<35 mm, <50% body depth) with longer and thicker screws (≥40 mm, >70% body depth). Both groups achieved adequate early restoration of vertebral height and vertebral compression angle, with no significant differences in postoperative correction or loss of reduction. At final follow-up, preservation of posterior vertebral height was slightly superior in the long-screw group, although no differences were observed in clinical outcomes, operation time, fluoroscopy use, or implant failure rates [17]. However, further evidence remains scarce.

### 4.3. Our Investigation

In this context, the study aimed to evaluate the influence of intermediate screw penetration depth on radiographic and clinical outcomes following short-segment posterior fixation for thoracolumbar junction burst fractures. Patients were stratified according to relative screw penetration (<50% versus ≥50% of vertebral body depth). At 12 months, both groups demonstrated significant improvements in kyphotic deformity (KD), anterior vertebral body height (AVBH), segmental kyphosis (SK), and sagittal index (SI), without statistically significant between-group differences. Functional outcomes assessed using ODI and VAS similarly improved in both cohorts, with no detectable intergroup differences.

Within the limits of this retrospective design, intermediate screw penetration depth was not independently associated with differences in radiographic alignment or patient-reported outcomes at 12 months. These findings suggest that screw penetration depth, when used within a standardized six-screw construct, may not represent a dominant determinant of short-term clinical or radiographic performance.

Potential technical advantages of shorter screws include reduced risk of anterior vertebral wall perforation, avoidance of severely comminuted anterior bone, and simplified insertion. However, these considerations remain procedural rather than outcome-based observations. Adjusted multivariable and mixed-effects analyses did not demonstrate a statistically significant association between screw penetration depth and primary radiographic endpoints. Although mixed-effects modeling including surgeon as a random effect did not reveal significant clustering, residual confounding related to surgical philosophy or intraoperative nuances cannot be entirely excluded.

The study was not designed or powered as an equivalence or non-inferiority trial. The reported precision estimates reflect statistical variability within the sample and should not be interpreted as predefined thresholds of clinical relevance. No minimal clinically important difference for kyphotic deformity was established a priori; therefore, conclusions regarding equivalence cannot be drawn.

From a biomechanical perspective, pedicle screw number, orientation, insertion depth, and diameter may influence construct stiffness and resistance to correction loss. Because these parameters were standardized across groups, their potential impact was minimized within this cohort. Accordingly, the present findings should be interpreted as exploratory and hypothesis-generating rather than definitive.

Recent literature published within the last five years continues to report heterogeneous results regarding construct configuration and screw characteristics in thoracolumbar fracture stabilization, underscoring the absence of clear consensus on optimal screw penetration strategies [11,25,26,27].

### 4.4. Limitations

The present study has several limitations that should be acknowledged to appropriately contextualize its findings. First, its retrospective design inherently limits the level of evidence and introduces potential selection bias, as treatment allocation and perioperative management were not prospectively controlled.

An important limitation concerns the non-randomized allocation of intermediate screw length. Screw selection followed long-standing team-specific surgical protocols rather than case-by-case randomization. Although all procedures were performed by two experienced spine surgeons and mixed-effects modeling including surgeon as a random effect did not demonstrate significant clustering, residual confounding related to surgeon-specific preferences, technical nuances, or intraoperative decision-making cannot be entirely excluded.

Although screw length was reported in absolute millimeters for descriptive clarity, group classification was based on relative vertebral body penetration (<50% vs. ≥50%) measured as a percentage of the anteroposterior vertebral body diameter. This approach accounts for anatomical variability in vertebral size; however, individual anatomical differences may still introduce subtle heterogeneity that cannot be completely eliminated in a retrospective design.

Another relevant limitation relates to bone quality assessment. Bone mineral density was not systematically evaluated using dual-energy X-ray absorptiometry (DEXA). While no documented history of metabolic bone disease was identified in the available medical records, undiagnosed osteoporosis or osteopenia cannot be entirely excluded. Therefore, residual confounding related to bone quality may be present and could potentially influence construct stability and radiographic outcomes.

The mechanism of injury was recorded from emergency department documentation and categorized as high- or low-energy trauma. Although fracture morphology was consistent with AO Spine A3–A4 burst patterns, the absence of systematic densitometric assessment limits definitive exclusion of fragility-related contributions in selected cases.

Although strict inclusion and exclusion criteria were applied, these resulted in a relatively homogeneous cohort of neurologically intact patients with single-level thoracolumbar junction burst fractures treated through an open posterior approach. Consequently, the generalizability of our findings is limited, and conclusions cannot be extended to elderly or clearly osteoporotic populations, patients with polytrauma or multilevel injuries, minimally invasive techniques, or alternative stabilization strategies.

A further methodological constraint concerns radiographic assessment. Baseline evaluation relied on CT imaging, whereas follow-up measurements were performed using standardized standing lateral radiographs. Despite magnification correction and excellent inter-rater reliability, this approach may introduce measurement variability, particularly in cases with borderline deformity values. Radiographs may also lack sensitivity in detecting subtle changes in alignment or early loss of correction.

The restriction of the primary radiographic endpoint to 12 months represents another limitation. While this strategy avoided attrition bias associated with incomplete long-term imaging, it may not capture late mechanical complications, progressive sagittal imbalance, or adjacent segment degeneration. Longer follow-up would be required to determine whether screw penetration depth influences outcomes beyond the first postoperative year.

Additionally, the study may be underpowered to detect differences in rare complications. Events such as implant failure or infection occurred infrequently, resulting in wide confidence intervals and limiting the precision of comparative estimates. Functional assessment was restricted to pain (VAS) and disability (ODI); broader quality-of-life metrics, return-to-work rates, and activity levels were not evaluated and may provide a more comprehensive functional perspective.

Finally, as a single-center investigation, the findings may reflect institutional preferences, specific implant systems, and local perioperative protocols. Although this consistency enhances internal validity, it may limit external applicability to centers employing different surgical philosophies or instrumentation systems.

### 4.5. Future Directions

Future research should aim to clarify the role of intermediate screw length through prospective, adequately powered studies with standardized screw selection criteria and longer-term radiological follow-up. Randomized or stratified trial designs would help mitigate confounding by surgeon preference and allow more precise estimates of clinically meaningful differences. Advanced imaging modalities, such as postoperative and follow-up CT scans, may improve the accuracy of detecting subtle alignment changes and implant-related complications. Additionally, incorporating broader patient-reported outcomes, return-to-work metrics, and cost-effectiveness analyses would provide a more comprehensive understanding of the clinical impact of different screw strategies. Finally, extending investigations to osteoporotic, elderly, and polytrauma populations, and to minimally invasive techniques, would enhance generalizability and guide personalized decision-making in the management of TLJ burst fractures.

## 5. Conclusions

In this retrospective cohort of patients with thoracolumbar burst fractures treated with short-segment posterior fixation including intermediate screws, intermediate screw penetration depth (<50% versus ≥50% of vertebral body depth) was not associated with statistically significant differences in radiographic alignment, functional outcomes, operative parameters, or complication rates at 12 months.

These findings should be interpreted within the exploratory nature and structural limitations of the study, including non-randomized allocation and the absence of systematic bone mineral density assessment. The present results do not establish equivalence and do not support definitive conclusions regarding the superiority of one screw penetration strategy over the other.

Further prospective, adequately powered studies with longer follow-up are required to clarify the biomechanical and clinical implications of intermediate screw penetration depth across broader patient populations.

## Figures and Tables

**Table 1:** Overview of Study Design (schematic summary).

Domain	Description
Study design	Retrospective cohort study (2019–2022), conducted according to STROBE guidelines. Not designed as equivalence or non-inferiority trial.
Setting	Single tertiary referral center; two senior surgical teams (>30 years’ experience each).
Population screened	212 trauma patients with suspected TLJ fractures (T11–L2).
Included patients	105 patients with AO Spine A3–A4 burst fractures, surgically treated with short-segment posterior fixation including two intermediate screws.
Exclusion criteria	Non-traumatic pathology, severe head injury, osteoporosis (T-score ≤–2.5), prior spine surgery, multiple fractures, PMMA augmentation, neurological deficits.
Group allocation	Based on *penetration ratio* measured on CT/X-ray. Group A (n = 56): screw length <50% vertebral body (35–40 mm). Group B (n = 49): screw length ≥50% vertebral body (≥45 mm). Allocation reflected long-standing team-specific protocols (not case-by-case).
Radiographic analysis	KD, SK, AVBH, SI measured on CT (baseline) and X-rays (follow-up). Vertebral body depth measured on midsagittal CT. Three blinded senior raters. ICC = 0.92 (95% CI 0.88–0.96).
Clinical outcomes	ODI, VAS, perioperative complications. Frankel grade documented pre/postoperatively; no new deficits observed.
Follow-up schedule	1, 3, 6, and 12 months clinical; 12-month imaging available for all patients (primary endpoint).
Postoperative protocol	Mobilization on POD2; rigid 3-point brace × 3 months.
Primary outcomes	Kyphotic deformity correction (KD), loss of correction (ΔKD, ΔSK, ΔSI) at 12 months.
Secondary outcomes	ODI, VAS, operative time, LOS, implant failure, complications (with 95% CI).
Statistical methods	Mann–Whitney U, Wilcoxon test, mixed-effects models (surgeon as random effect), ICC, effect sizes (Cohen’s d, HL median difference, rank-biserial). Significance *p* < 0.05.
Missing data	None (complete-case analysis).
Sample size justification	No a priori power calculation. Post hoc precision analysis: KD differences estimated within ±2.1° (95% CI).
Software	IBM SPSS Statistics for Windows, Version 26.0 (IBM Corp., Armonk, NY, USA).

AVBH: Anterior Vertebral Body Height; CI: Confidence Interval; CT: Computed Tomography; ICC: Intraclass Correlation Coefficient; KD: Kyphotic Deformity; LOS: Length of Stay; ODI: Oswestry Disability Index; PMMA: Polymethyl Methacrylate; POD: Postoperative Day; SI: Sagit-tal Index; SK: Segmental Kyphosis; SPSS: Statistical Package for the Social Sciences; STROBE: STrengthening the Reporting of OBservational studies in Epidemiology; TLJ: Thoracolumbar Junction; VAS: Visual Analog Scale.

**Table 2:** Baseline demographic and clinical characteristics of patients in Group A (<50% vertebral body penetration) and Group B (≥50% vertebral body penetration).

Demographics	Group A	Group B	*p* Value
n° of patients	56	49	
Age	56.4 (+/− 15.4)	55.8 (+/− 16.2)	0.817
Sex	M:41; F:15	M:37; F:12	
BMI	26.9 +/− 3.1	27.6 +/− 3.8	0.621
Diabetes	3 (5.3%)	5 (10.2%)	0.724
Smokers	26 (46.2%)	24 (49%)	0.836
Other comorbidities	21 (37.4%)	23 (46.9%)	0.792
Type of fracture (AO classification)			
A3	39 (69.6%)	33 (67.3%)	0.554
A4	17 (30.4%)	16 (32.7%)	0.607
Fractured Level			
D11	6 (10.7%)	5 (10.1%)	0.819
D12	20 (35.6%)	19 (38.8%)	0.724
L1	18 (32.1%)	16 (32.6%)	0.659
L2	12 (21.6%)	9 (18.5%)	0.718
Traumatic mechanism			
Traffic accident	32 (56.7%)	29 (59.2%)	0.323
High falling injury	14 (24.9%)	12 (24.5%)	0.534
Other causes	10 (18.4%)	8 (16.3%)	0.821
Follow up	27.2 (+/− 10.2) m	28.1 (+/− 9.4) m	0.619

**Table 3:** Radiographic data.

Radiographic Parameters	Group A	Group B	* p * Value
n° of patients	56	49	
Preoperative KD	17.4 (+/− 5.1)°	18.1 (+/− 4.3)°	0.278
Postoperative KD	3.2 (+/− 4.2)°	3.6 (+/− 6.1)°	0.413
*p*	0.0001	0.0002	
12 m FU KD	4.4 (+/− 4.5)°	4.1 (+/− 5.6)°	0.312
*p* (vs. pre-op)	0.0021	0.0029	
Preoperative AVBH	14.6 (+/− 9.1) mm	14.1 (+/− 8.2) mm	0.394
Postoperative AVBH	22.3 (+/− 5.8) mm	23.4 (+/− 6.3) mm	0.723
*p*	0.0017	0.0021	
12m FU AVBH	21.9 (+/− 7.3) mm	22.2 (+/− 8.1) mm	0.846
*p* (vs. pre-op)	0.0031	0.0037	
Preoperative SK	13.8 (+/− 6.1)°	12.7 (+/− 5.9)°	0.492
Postoperative SK	6.9 (+/− 5.2)°	7.3 (+/− 5.8)°	0.378
*p*	0.0042	0.0034	
12m FU SK	7.7 (+/− 4.9)°	7.2 (+/− 5.1)°	0.477
*p* (vs. pre-op)	0.0059	0.0042	
Preoperative SI	22.4 (+/− 4.1)°	22.8 (+/− 3.8)°	0.381
Postoperative SI	7.4 (+/− 3.9)°	6.7 (+/− 1.9)°	0.613
*p*	0.0002	0.0004	
12m FU SI	7.2 (+/− 4.3)°	7.1 (+/− 3.6)°	0.732
*p* (vs. pre-op)	0.0012	0.0031	

**Table 4:** Surgical data.

Surgical Parameters	Group A	Group B	* p * Value
n° of patients	56	49	
Operative time	92 (+/− 23) min	94 (+/− 18) min	0.921
Fluoroscopy number	6.3 (+/− 2.4)	6.9 (+/− 3.1)	0.719
Type of instrumentation			
Expedium	24 (42.7%)	21 (42.8%)	0.935
CD Solera	32 (57.3%)	28 (57.2%)	0.891
LOS (days)	4.2 (+/− 1.1)	4.7 (+/− 0.8)	0.383

LOS: Length of stay.

**Table 5:** Clinical outcomes and complications.

Clinical Outcomes	Group A	Group B	* p * Value
n° of patients	56	49	
Preoperative VAS	7.4 (+/2.2)	7.7(+/− 1.9)	0.475
Postoperative VAS	6.9 (+/− 2.9)	6.5 (+/− 2.1)	0.161
1 month FU VAS	4.1 (+/− 1.7)	4.5(+/− 1.1)	0.224
6 months FU VAS	3.8 (+/− 1.3)	4.1 (+/− 1.8)	0.614
12 months FU VAS	2.9 (+/− 1.8)	2.1(+/− 0.8)	0.145
Preoperative ODI	83.9 (+/− 15.2)	84.1 (+/− 17.2)	>0.05
Postoperative ODI	71.8 (+/− 15.6)	74.6 (+/− 18.8)	>0.05
1 month FU ODI	59.2 (+/− 13.4)	61.3 (+/− 14.2)	>0.05
6 months FU ODI	33.6 (+/− 12.3)	35.1 (+/− 11.4)	>0.05
12 months FU ODI	22.4 (+/− 9.7)	18.9 (+/− 8.9)	>0.05
**Complications**			
Screws loosening	2 (3.6%)	1 (2%)	0.223
Implant failure	1 (1.8%)	1(2%)	0.421
Wound infection	1 (1.8%)	1 (2%)	0.245

## Data Availability

The datasets generated and/or analyzed during the current study are not publicly available due to institutional data protection policies but are available from the corresponding authors upon reasonable request.

## Abbreviations

The following abbreviations are used in this manuscript:

AO	Arbeitsgemeinschaft für Osteosynthesefragen (AO Spine classification)
AVBH	Anterior vertebral body height
CT	Computed tomography
ICC	Intraclass correlation coefficient
IRR	Inter-rater reliability
KD	Kyphotic deformity
LOS	Length of stay
ODI	Oswestry Disability Index
PACS	Picture Archiving and Communication System
SI	Sagittal index
SK	Segmental kyphosis
TLJ	Thoracolumbar junction
VAS	Visual Analog Scale
WMA	World Medical Association
